# The influence of hypoxia in predicting COVID-19 mortality in patients with kidney dysfunction on admission in 2 centers in sub-Saharan Africa

**DOI:** 10.1080/0886022X.2026.2719962

**Published:** 2026-08-26

**Authors:** Yannick Mayamba Nlandu, Jean-Robert Rissassi Makulo, Yannick Mompango Engole, Ben Izizag Bepouka, Marie-France Ingole Mboliasa, Aliocha Natuhoyila Nkodila, Augustin Luzayadio Longo, François Musungayi Kajingulu, Hippolyte Nani-Tuma Situakibanza, Vieux Momeme Mokoli, Justine Busanga Bukabau, Nazaire Mangani Nseka, Marie Essig, Ernest Kiswaya Sumaili

**Affiliations:** ^a^ Nephrology Unit, University Hospital of Kinshasa, University of Kinshasa, Kinshasa, Democratic Republic of Congo; ^b^ Intensive Care Unit, Centre Médical de Kinshasa, Kinshasa, Democratic Republic of Congo; ^c^ Secrétariat Technique du Comité Multisectoriel de la Riposte Contre la Covid-19, Kinshasa, Democratic Republic of the Congo; ^d^ Unit of Infectious and Tropical Diseases, University Hospital of Kinshasa, Kinshasa, Democratic Republic of the Congo; ^e^ Department of Family Medicine and Primary Care, Protestant University in Congo, Kinshasa, Democratic Republic of Congo; ^f^ Nephrology Department, Ambroise Paré Hospital, AP-HP University Paris-Saclay, Boulogne-Billancourt, France

**Keywords:** COVID-19, kidney dysfunction, hypoxia, survival, organ cross talk

## Abstract

Kidney dysfunction (KD) and hypoxia on admission are separately associated with COVID-19 mortality. Estimation of GFR using creatinine and peripheral oxygen saturation (SpO2) may be useful for stratification of at-risk populations. We performed a two centers retrospective cohort study of 359 COVID-19 patients aged 18 years or older to explore the impact of admission kidney function and hypoxia in predicting COVID-19 mortality. KD at admission was defined as estimated glomerular filtration rate (eGFR) calculated by CKD EPI < 60 mL /min/1.73 m^2^ using creatinine and hypoxia as a SpO2 ˂ 90%. Survival (time-to death) curves were built using the Kaplan–Meier methods. Association between mortality, hypoxia and KD was evaluated by multivariate Cox regression models with a significance level of the p-value set at 0.05. There were 152 patients (42.3%) with an eGFR < 60 mL/min/1.73 m^2^ and 208 patients (74.8%) with hypoxia on admission. Overall in-hospital mortality was 27.6%, rising to 59.7% in patients with eGFR below 60 mL/min and hypoxia (*p* < 0.001). Survival was lower in the group of patients with a eGFR below 60 mL/min/1.73 m^2^ and hypoxia (*p* < 0.001). Predictors of mortality were eGFR ≥ 60 mL/min/1.73 m^2^ with hypoxia (HR 2.05 [1.01–4.16], *p* = 0.048), eGFR < 60 mL/min/1.73 m^2^ without hypoxia (HR 3.20 [1.73–5.94], *p* < 0.001) and eGFR < 60 mL/min/1.73 m^2^ with hypoxia (HR 6.55 [3.63–11.84], *p* < 0.001). KD on admission and hypoxia are common in COVID-19. Its association with in-hospital mortality should encourage its use in the stratification of patients at risk of death. Influence of hypoxia could suggest a pattern of kidney-lung cross talk.

## Introduction

COVID-19 has been widely described, with the main aim of determining mortality risk factors in view of the large number of deaths worldwide. At a very early stage, emphasis was placed on renal parameters, in particular the glomerular filtration rate (GFR) on admission [1]. Although widely used as a marker of kidney function, estimated glomerular filtration rate (eGFR) based on serum creatinine levels remains a challenge in the context of acute illness. In fact, it may reflect baseline kidney function, or either progressive or resolving kidney damage. Nonetheless, it remains useful, on the one hand, for adapting drugs and, on the other, for stratifying patients at risk of mortality. Several studies have analyzed the association between eGFR at admission and COVID-19. These studies have reported a high risk not only of contracting COVID-19 in kidney failure patients, but also of presenting a severe form of the disease, with longer hospital stays and more complications (mechanical ventilation, dialysis, death), gradually increasing in advanced stages of kidney function impairment [2]. Indeed, the associated impairment of innate and adaptive immunity may explain the increased susceptibility of these patients to infections [3]. The relationship between COVID-19 and kidney damage can also be seen in the sense that kidney damage secondary to COVID-19 may exist [4].

Irrespective of the cause of admission, kidney failure can impact disease progression in several ways. Kidney failure may be responsible for an imbalance in the COVID-19 patient’s fluid, electrolyte and acid-base status. Kidney failure also leads to a pro-inflammatory and immunosuppressive state, which promotes distant damage to several organs, including the lungs, well known as ‘organ cross-talk’, leading to hypoxemia and hypoxia [5,6].

Kidney and respiratory failures are largely reported as a common associated complication, which independently lead to higher mortality among in COVID-19 patients. This in-hospital mortality is increased when the two conditions overlap. In the Democratic Republic of Congo, although the association between kidney and respiratory failure has been demonstrated [7], the clinical outcomes of the simultaneous dysfunction of the kidneys and the lungs, a specific feature of the COVID-19 patients admitted to the intensive care units have not been clearly reported. The aim of the present study was to assess the impact of kidney dysfunction of COVID-19 patients at admission on mortality and to evaluate the association between hypoxia, reduction of kidney function and risk of death in patients hospitalized for COVID-19.

## Methods

This observational, retrospective cohort study was carried out in Cliniques Universitaires de Kinshasa (CUK) and Center Médical de Kinshasa (CMK), two COVID-19 centers designated by the DRC Ministry of Health in Kinshasa between March 27, 2020 and January 27, 2022. These centers (public [CUK] and private [CMK]) previously described had an intensive care unit with multidisciplinary medical intervention [8,9]. These centers were also the only ones in the city of Kinshasa to offer an acute dialysis service for COVID-19 patients during the two first COVID-19 waves, with intermittent hemodialysis as the only form of renal replacement therapy (RRT) modality. Patients were treated according to the recommendations of the national protocol for the management of COVID-19, with hydroxychloroquine and azithromycin as first-option treatment [10,11]. Corticosteroids and heparin (prophylactic or curative dose) were used according to the severity of COVID-19 disease [11]. Management of respiratory failure includes conventional oxygen therapy, noninvasive ventilation (high-flow nasal cannula oxygen therapy (HFNC), continuous positive airway pressure (CPAP) and noninvasive ventilation (NIV)) and invasive mechanical ventilation. Sampling was exhaustive and consecutive. All symptomatic patients aged ≥18 years with a positive COVID-19 test by real-time reverse transcriptase-polymerase chain reaction (RT-PCR) were included. Patients with a hospital stay of less than 72 h, those without an admission serum creatinine measurement (SCr) and those on chronic hemodialysis were excluded from the study.

Sociodemographic data (age, gender), patient comorbidities (hypertension, diabetes mellitus, obesity, Chronic Kidney Disease: CKD, human immunodeficiency virus infection: HIV, heart failure: HF), vital signs (blood pressure [BP], respiratory rate [RR], heart rate [HR], temperature, peripheral oxygen saturation [SpO_2_]) were collected on admission. Biological data on admission included hemoglobin [Hb], hematocrit [Htc], white blood cells [WBC], C-reactive protein [CRP], Aspartate aminotransferase [AST], alanine aminotransferase [ALT], blood urea nitrogen [BUN] and creatinine). Patient severity was reported according to the fifth edition of Guidelines on the Diagnosis and Treatment of COVID-19(12). In addition, we collected information about the treatment received and/or complications (administration of corticosteroids, mechanical ventilation [MV] or hemodialysis) and outcomes during the hospital admission. Kidney dysfunction at admission was defined as eGFR < 60 mL/min/1.73 m^2^ [1]. GFR was estimated using the CKD-Epi (Chronic Kidney Disease Epidemiology Collaboration) formula without using the race factor [13]. The eGFR was categorized into two categories (eGFR <60 mL/min/1.73 m^2^ and eGFR ≥ 60 mL/min/1.73 m^2^). Hypoxia was defined as a peripheral oxygen saturation ˂90% and correspond to severe form of COVID-19 according the WHO definition. Chronic kidney disease (CKD) was identified based on the patient’s medical history. The primary outcome was the in-hospital mortality. Survival defined as time to death and the vital status until the 48th day. The severity of COVID-19 was categorized according the WHO classification [14].

## Statistical analysis

Data were compiled in an Excel 2010 database of patients followed for COVID-19. Categorical variables were presented as frequencies based on the total number of non-missing values, and quantitative variables, depending on whether they were normally distributed or not, as mean ± standard deviation or median-interquartile range. The Kolmogorov–Smirnov test was used to define the normality of the sample distribution. The entire group was stratified according to eGFR and hypoxia status at admission. The one-way ANOVA with *post hoc* Bonferroni test, the Kruskall–Wallis and Pearson chi-square test or the Fischer exact test were used to compare the means, medians and proportions in more than two groups, as appropriate. Survival was analyzed by Kaplan–Meier (time to death). Patients who survived to the end of the study or were transferred to another center were censored. Survival curves were compared using the Log Rank test. A multivariable Cox regression model was used to identify the independent factors associated with mortality in general and the association of hypoxia and eGFR with death in particular. Factors associated with mortality in unadjusted univariable cox regression were included in a multivariable cox regression model to identify independent factors associated with mortality, the odds ratio (OR) was calculated for each independent variable. We excluded variables from the univariable analysis if their between-group differences were not significant, if the number of events was too small to calculate odds ratios. Only significant variables in univariable cox regression were retained in the final model. The variance inflation factor (VIF) coefficient was used to identify multicollinearity between factors. Two multivariate mathematical models were used to limit the cumulative effect of independent variables. The threshold of statistical significance was *p* ˂ 0.05. Statistics were performed with IBM SPSS for Windows version 24.

## Ethics approval

To ensure the independence of the assessment, the research was approved by the National Ethics Committee of the Democratic Republic of Congo (N°225/CNES/BN/PMMF/2020). Data were collected anonymously and confidentially. Due to the retrospective nature of the study, the National Ethics Committee of the Democratic Republic of Congo waived the need to obtain informed consent. All methods were performed in accordance with the principles of the Declaration of Helsinki.

## Results

**Figure 1.**
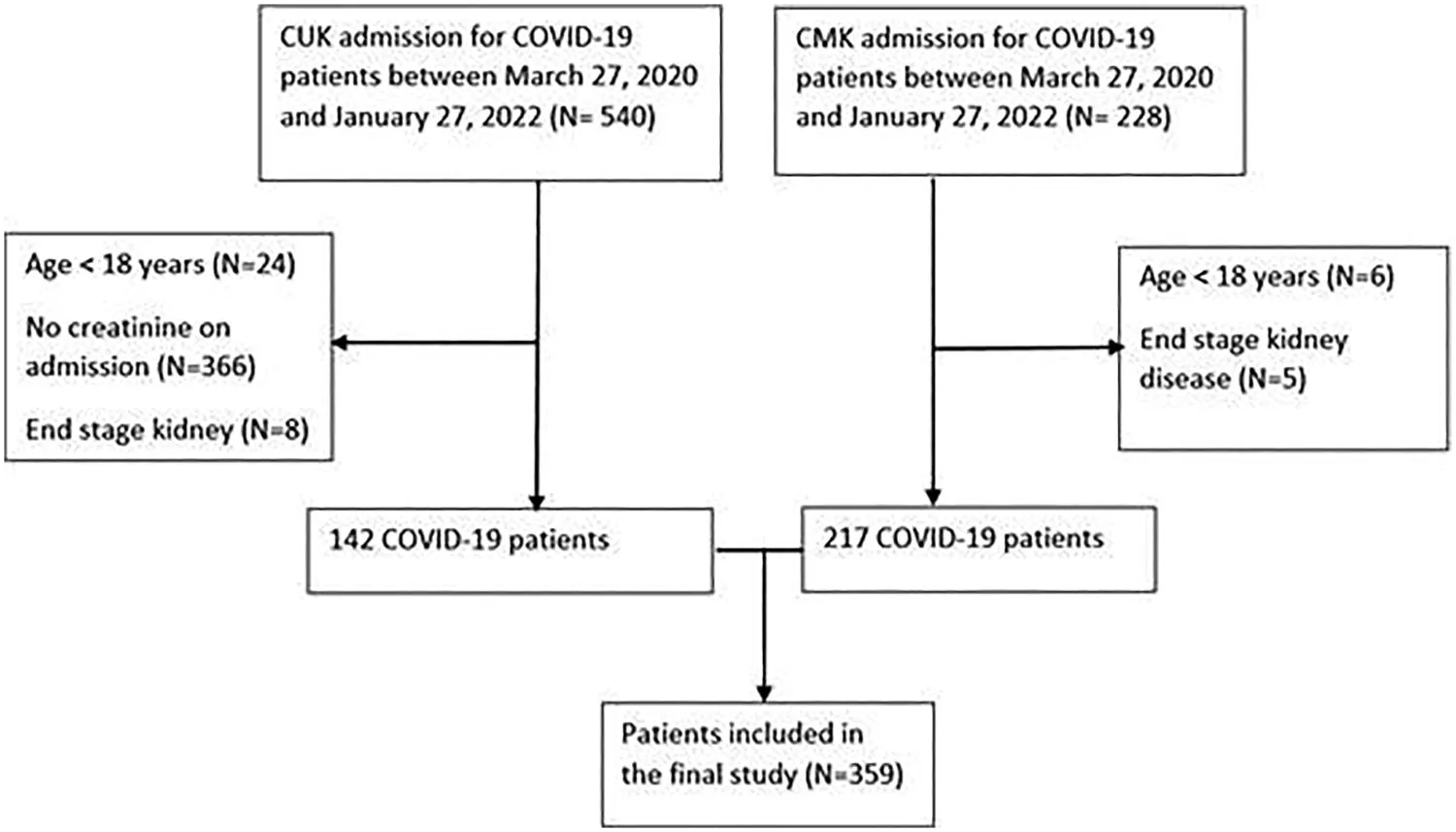
Flow chart of study population selection.

**Figure 2.**
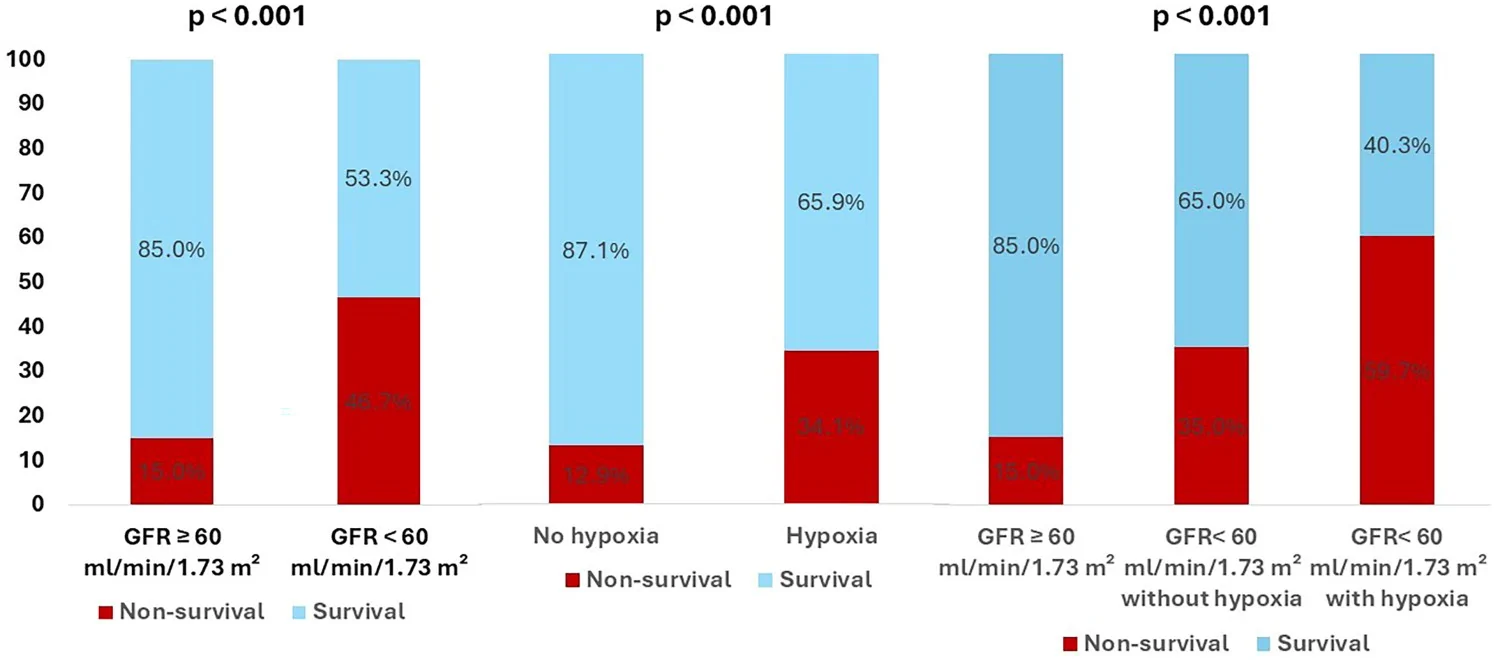
Mortality rate according eGFR (a), Hypoxia (b) and Hypoxia + eGFR (c) status.

Three hundred and fifty-nine patients were included in the present study (Figure 1), with a mean age of 59.2 ± 14.6 years, and were predominantly male (75.2%), black (88%) and hypertensive (50.1%). The mean peripheral oxygen saturation (SpO_2_) and eGFR of the study population was, respectively 88.8 ± 11.8% and 62.9 ± 31.7 mL/min/1.73 m^2^. One hundred fifty-two out of 359 patients (42.3%) had an admission eGFR < 60 mL/min/1.73 m^2^ and 29 out of 359 (8.1%) patients received acute hemodialysis treatment with the main indications being anuria (83.3%), hypervolemia (75%), hyperkalemia (50%) and uremic encephalopathy (16.7%). Seventy-two patients out of 359 patients (20.05%) had an admission eGFR < 60 mL/min with hypoxia. The characteristics of the patients, stratified by eGFR and hypoxia status are shown in Table 1. Overall in-hospital mortality was 27.6%, rising to 46.7% in patients with eGFR below 60 mL/min/1.73 m^2^ (*p* ˂ 0.001), 59.7% in patients with eGFR < 60 mL/min/1.73 m^2^and hypoxia (*p* ˂ 0.001) (Figure 2). Compared to the patients with an admission eGFR > 60 mL/min/1.73 m^2^, these patients had an age greater than or equal to 65 years (51.4% vs 30.4%, *p* = 0.001), at least one comorbidity (77.8% vs 54.6%, *p* = 0.002) with hypertension as the most important (69.4% vs 42.5%, *p* ˂ 0.001), lower SpO_2_ (86.1 ± 12.5 vs 90.0 ± 11.0, *p* = 0.028), higher systolic BP (146.5 ± 26.4 vs 135.4 ± 17.9, *p* = 0.005). These patients presented an inflammatory profile with a higher AST/ALT ratio of 1.2 [1.1–1.8] vs. 1.06 [0.72–1.37], *p* = 0.002). Kidney parameter as BUN (76.8 [33.0–96.0] vs 24.0 [20.1–36.0], *p* < 0.001), creatinine (1.6[1.0–2.2] vs 0.88[0.74–1.13], *p* = 0.001) and sodium (140.4 ± 9.8 vs 3.8 ± 0.5, *p* = 0.011) were higher in the group of patients with a eGFR on admission < 60 mL/min/1.73 m^2^and hypoxia. They were black (97.2% vs 83.6%, *p* = 0.005) and had a higher use of corticosteroid (38.1% vs 12.4%, *p* = 0.001) and anticoagulation (41.7% vs 24.8%) therapy.

**Table 1.** Baseline demographics, severity-of-illness parameters, and treatment during hospital admission stratified by vital status.

Variables	All (*n* = 359)	eGFR ≥ 60 mL/min/1.73 m² (*n* = 207)	eGFR< 60 mL/min/1.73 m² + No hypoxia (*n* = 80)	eGFR < 60 mL/min/1.73 m² + Hypoxia (*n* = 72)	*p*
**Sociodemographic parameters**					
Age, years	59.2 ± 14.6	56.4 ± 14.2	62.9 ± 15.3	63.2 ± 13.3	**<0.001**
Age ≥ 65, n (%)	138 (38,4)	63 (30,4)	38 (47,5)	37 (51,4)	**0.001**
Sexe, males, n (%)	270 (75,2)	154 (74,4)	64 (80,0)	52 (72,2)	0.494
Race African ancestry, n(%)	316 (88,0)	173 (83,6)	73 (91,3)	70 (97,2)	**0.005**
Comorbidities	218 (60,7)	113 (54,6)	49 (61,3)	56 (77,8)	**0.002**
Hypertension, n (%)	180 (50,1)	88 (42,5)	42 (52,5)	50 (69,4)	**<0.001**
Diabetes, n (%)	82 (22,8)	45 (21,7)	15 (18,8)	22 (30,6)	0.191
CKD, n (%)	5 (1,4)	1 (0,5)	2 (2,5)	2 (2,8)	0.131
Heart Failure, n (%)	20 (5,6)	6 (2,9)	6 (7,5)	8 (11,1)	**0.021**
HIV, n (%)	5 (1,4)	3 (1,4)	0 (0,0)	2 (2,8)	0.307
Obesity, n (%)	41 (11,4)	23 (11,1)	10 (12,5)	8 (11,1)	0.948
**Baseline medications**					
Diuretics, n(%)	32 (14.7)	20 (13.8)	10 (19.6)	2 (9.5)	0.499
Beta blockers, n (%)	21 (9.7)	9 (6.2)	10 (19.6)	2 (9.5)	0.023
ACE inhibitors, n (%)	27 (12.4)	15 (10.3)	10 (19.6)	2 (9.5)	0.229
CCBs, n (%)	79 (36.4)	45 (31.0)	23 (45.1)	11 (52.4)	0.054
ARBs, n (%)	20 (9.2)	9 (6.2)	6 (11.8)	5 (23.8)	0.056
**Parameters at admission**					
Body temperature (C)	37.1 ± 0.9	37.0 ± 0.84	37.3 ± 0.9	37.0 ± 1.1	0.219
RR/minutes	27.8 ± 6.5	27.3 ± 6.4	26.9 ± 5.5	31.0 ± 7.5	**0.002**
SpO_2_ %	88.8 ± 11.8	90.0 ± 11.0	87.2 ± 12.7	86.1 ± 12.5	**0.028**
SBP, mmHg	138.3 ± 20.6	135.4 ± 17.9	140.3 ± 21.3	146.5 ± 26.4	**0.005**
DBP, mmHg	83.1 ± 13.9	82.6 ± 13.0	84.3 ± 15.1	83.6 ± 15.8	0.673
MAP, mmHg	101.1 ± 15.8	99.6 ± 15.2	103.0 ± 15.9	104.5 ± 17.4	0.106
Heart rate, bpm	87.6 ± 14.8	86.8 ± 14.0	88.2 ± 16.7	89.9 ± 15.0	0.451
**COVID-19 severity**					**<0.001**
Mild, n (%)	68 (18,9)	38 (18,4)	30 (37,5)	0 (0,0)	
Moderate, n (%)	193 (53,8)	114 (55,1)	40 (50,0)	39 (54,2)	
Severe/critical, n (%)	98 (27,3)	55 (26,6)	10 (12,5)	33 (45,8)	
**Medications during Hospitalization**					
Corticosteroids, n (%)	43 (19,8)	18 (12,4)	17 (33,3)	8 (38,1)	**0.001**
Anticoagulants, n (%)	89 (24,8)	39 (18,8)	20 (25,0)	30 (41,7)	**0.001**
**Complications**					
Hemodialysis, n (%)	29 (8,1)	15 (7,2)	7 (8,8)	7 (9,7)	0.803
Mechanical ventilation, n (%)	20 (5,6)	7 (3,4)	6 (7,5)	7 (9,7)	0.120
**Biological parameters**					
Hb, g/dl	13.4 ± 2.8	13.7 ± 2.3	12.9 ± 2.3	12.8 ± 4.1	0.045
Htc,%	39.2 ± 8.3	40.1 ± 7.6	38.1 ± 6.8	37.7 ± 11.7	0.084
Na+, mmol/L	137.7 ± 6.6	137.3 ± 5.1	137.2 ± 7.2	140.4 ± 9.8	**0.016**
K+, mmol/L	3.9 ± 0.6	3.9 ± 0.5	4.0 ± 0.8	4.0 ± 0.7	0.427
BUN, mg/dL	30.0 (21.6–40.8)	24.0 (20.1–36.0)	29.7 (12.9–76.2)	76.8 (33.0–96.0)	**<0.001**
Creatinin, mg/dl	1.03 (0.77–1.24)	0.88 (0.74–1.13)	1.24 (0.66–2.66)	1.6 (1.0–2.2)	**0.001**
DFG CKD-Epi	62.9 ± 31.7	85.1 ± 17.1	34.6 ± 18.8	30.4 ± 20.5	**<0.001**
WBC, count,× 10^3^/L	7440.0 (6540.0–9960.0)	7690.0 (3940.0–11280.0)	7195.0 (6540.0–10570.0)	11040.0 (7440.0–16720.0)	0.136
Neutrophils x10^3^/L	5122.4 (4179.0–6403.0)	4883.0 (2522.5–7370.5)	5046.4 (4568.2–9090.0)	6403.0 (4166.4–15884.0)	0.395
Lymphocyts x10^3^/L	1111.8 (985.0–1240.0)	1032.2 (879.0–1213.0)	1128.9 (845.0–2037.5)	2529.6 (501.6–3864.0)	0.156
Ratio N/L	4.9 (4.5–5.5)	4.9 (4.5-6 ;6)	4.7 (2.7–10.8)	4.9 (1.6–31.7)	0.086
CRP, mg/L	139.0 (107.0–218.0)	223.5 (108.0–336.4)	114.0 (36.0–152.0)	107.0 (63.0–204.0)	0.399
AST, U/l	48.0 (38.0–91.0)	49.5 (28.0–119.0)	68.0 (38.0–298.0)	48.0 (35.0–191.0)	**0.001**
ALT, U/l	45.0 (35.0–67.0)	46.0 (26.0–67.9)	46.5 (29.0–579.0)	44.0 (29.0–107.0)	0.080
AST/ALT ratio	1.09 (0.92–1.37)	1.06 (0.72–1.37)	1.38 (0.51–2.19)	1.20 (1.1–1.8)	**0.002**

Data are mean ± standard, median (IQR), n (%), or n/N (%). Percentage are based on the total number of non-missing values in each category and not necessarily on the total number of participants. p values were calculated by Mann–Whitney U test or χ^2^ test, as appropriate. ACE: angiotensin converting enzyme; ALT: alanine amino transferase; ARB: angiotensin receptor blockers; AST: aspartate amino transferase; CCB: calcium channel blockers; SpO_2_: peripheral oxygen saturation; Bpm: beats per minutes; CKD: chronic kidney disease; BUN: blood urea nitrogen; CRP: C-reactive protein; DBP: diastolic blood pressure; Hb: hemoglobin; HIV: human immunodeficiency virus; HF: heart failure; HR: heart rate; MAP: mean arterial pressure; MV: mechanical ventilation; RR: respiratory rate; WHO: world health organization.

**Figure 3.**
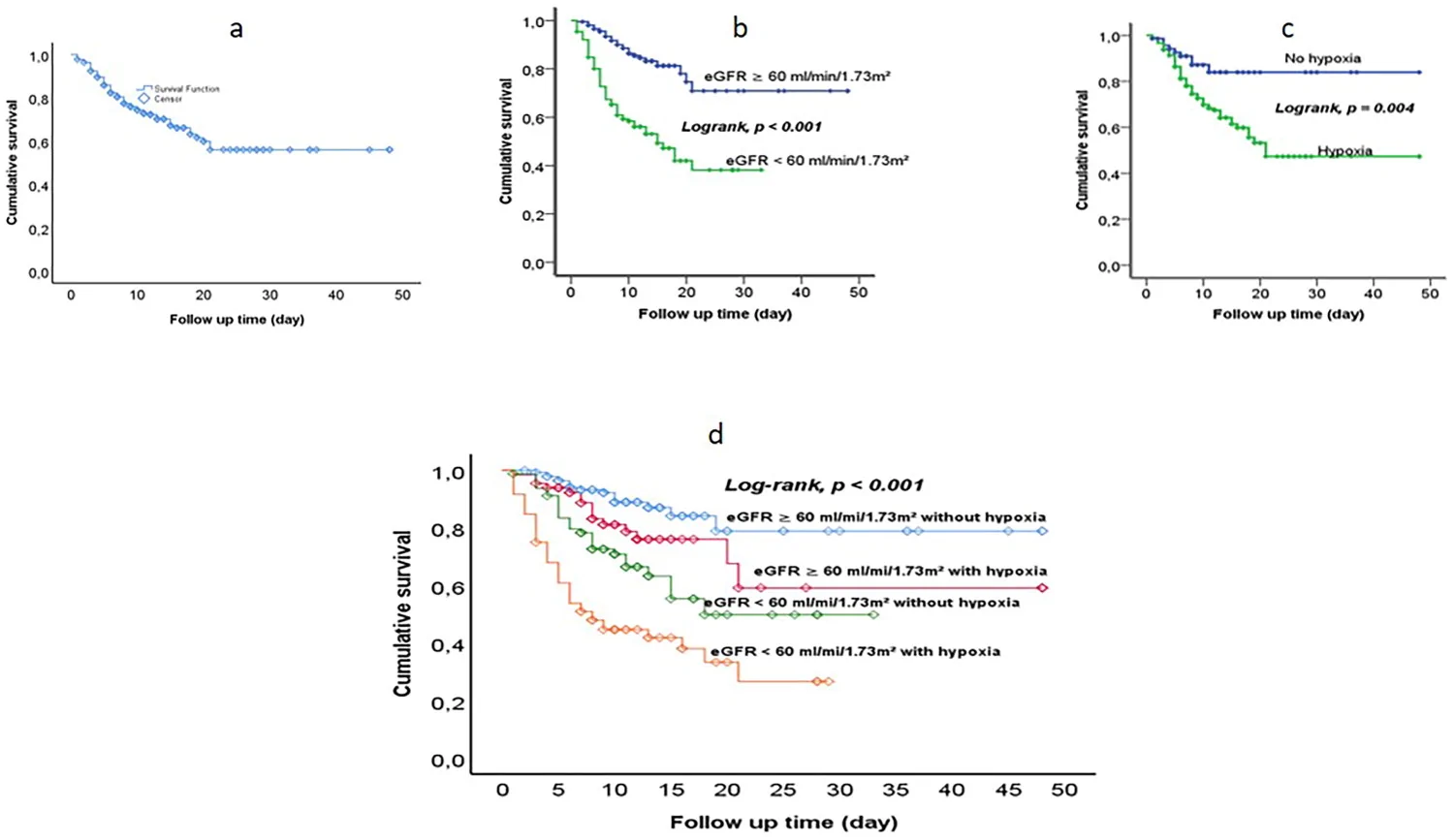
Survival of COVID-19 patients study population (a) according eGFR(b), hypoxia (c) and eGFR + hypoxia status (d).

Patients in nonsurvivors group had significantly higher age (64.6 ± 13 vs 57.1 ± 14.7), Systolic Blood Pressure (143.4 ± 22.7 vs 136.4 ± 19.4), Mean arterial pressure (104.5 ± 17.5 vs 99.9 ± 15) and lower SpO_2_ (83.5 ± 14.0 vs 90.9 ± 10.1), eGFR (40.0 ± 15.4 vs 72.0 ± 27.4). The patients in nonsurvivors group had also significantly more comorbidities (72.5% vs 56.0), hypertensive status (62.7% vs 45.1%), obesity (22.5% vs 7.0%). These patients present the severe form of COVID-19 (40.2% vs 22.2) and more complications requiring hemodialysis (18.6 vs 3.9%), use of vasopressors (57.9% vs 2%) and mechanical ventilation (71.7% vs 4.4). Predictors of mortality were eGFR ≥ 60 mL/min/1.73 m^2^ with hypoxia (HR 2.05 [1.01–4.16], *p* = 0.048), eGFR < 60 mL/min/1.73 m^2^ without hypoxia (HR 3.20 [1.73–5.94], *p* < 0.001) and eGFR < 60 mL/min/1.73 m^2^ with hypoxia (HR 6.55 [3.63–11.84], *p* < 0.001) (Table 2). Overall patient survival decreased with time, with 81.1% survival at Day 7, 74.4% at Day 14 and 71% at Day 21. Survival was lower in the group of patients with a eGFR below 60 mL/min/1.73 m^2^ with hypoxia (Figure 3). Median survival for patients with eGFR ≥60 mL/min/1.73 m^2^, ˂60 mL/min/1.73 m^2^ without hypoxia and ≤60 mL/min/1.73 m^2^ with hypoxia was 15 [10–21], 10 (8–11) and 7 (5–9) days, respectively. Survival was also lower (34.5% vs 74.8%, *p* ˂0.001) in the group requiring acute hemodialysis at 48 days.

**Table 2.** Predictors of mortality in COVID-19 patient.

	Univariate analysis		Multivariate analysis					
			Model 1		Model 2		Model 3	
Variables	*p*	HR (CI 95%)	*p*	aHR (CI 95%)	*p*	aHR (CI 95%)	*p*	aHR (CI 95%)
**Age ≥ 65years**								
No		1		1		1		1
Yes	0.009	1,.69 (1.14–2.49)	0.346	1.22 (0.81–1.82)	0.384	1.20 (0.80–1.79)	0.208	1.29 (0.87–1.93)
**Comorbidities**								
No		1		1				
1	0.108	1.51 (0.91–2.49)	0.289	1.31 (0.79–2.17)		–		–
≥2	0.001	2.27 (1.40–3.69)	0.065	1.61 (0.97–2.65)		–		–
**Hypertension**								
No						1		1
Yes	0.017	1.63 (1.09–2.44)		–	0.630	1.11 (0.73–1.69)	0.208	1.30 (0.86–1.96)
**Obesity**								
No		1				1		1
Yes	0.001	2.15 (1.35–3.43)		–	**0.007**	1.94 (1.19–3.15)	**0.013**	1.81 (1.13–2.89)
**Hypoxia + eGFR**								
eGFR ≥60 + no hypoxia		1		1		1		
eGFR ≥60 + hypoxia	0.048	2.03 (1.01–4.11)	0.067	1,94 (0.96–3.94)	**0.048**	2.05 (1.01–4.16)	–	–
eGFR < 60 + no hypoxia	<0.001	3.31 (1.79–6.12)	<0.001	3.14 (1.69–5.83)	**<0.001**	3.20 (1.73–5.94)	–	–
eGFR < 60 + hypoxia	<0.001	7.11 (4.00–12.63)	<0.001	6.16 (3.42–11.09)	**<0.001**	6.55 (3.63–11.84)	–	–
eGFR (mL/min/1.73 m²)								

Bold values are the statistically significant *p* defining the variables associated with mortality. eGFR: estimated glomerular filtration rate.

## Discussion

This retrospective study of 359 patients in two centers in sub-Saharan Africa shows that kidney function impairment on admission are common and that its association with mortality in patients with COVID-19 disease increases with hypoxia.

Kidney impairment in COVID-19 covers a wide spectrum of impairment, both functional and structural. It is in this context that proteinuria and hematuria have been reported, as well as reduced eGFR leading to acute kidney injury (AKI) and even dialysis [15]. Impairment of kidney function on admission may therefore reflect either baseline kidney function, and thus the existence of chronic kidney disease (CKD), a process of worsening kidney function, or rather a process of renal recovery, all of which are associated with mortality [16,17]. This hypothesis was well supported in the study by Zolotov et al. where the association between admission eGFR and mortality in ICU patients was reported according to their evolutionary profile based on admission creatinine and grouped into acute kidney injury (AKI) increase, AKI decrease and chronic kidney disease (CKD) [18]. These authors state that calculating the eGFR on admission can be an excellent tool for predicting mortality [18].

Impaired kidney function on admission of COVID-19 patients to hospital is common. Nearly 4 out of 10 patients in our cohort had impaired kidney function on admission. This high proportion may reflect the bidirectional relationship between COVID-19 and kidney impairment. On the one hand, a higher susceptibility to infection in CKD patients, and on the other, greater kidney damage associated with COVID-19. In our cohort, at least six out of 10 patients had a comorbidity (hypertension, diabetes mellitus) at risk of CKD. Indeed, lower eGFR is very common in multimorbid patients [19]. As in many studies [16,20,21], less than 10% of patients in our cohort had a history of CKD on admission, suggesting a possible direct causal relationship between COVID-19 and AKI [21].

The mortality associated with impaired kidney function is easy to understand. The accumulation of uremic toxins is conducive to a pro-inflammatory state, and the disruption of the fluid and sodium balance is conducive to a positive water balance, which can have harmful effects on the respiratory system, with an increase in hypoxemia and hypoxia. The imbalance in potassium homeostasis is conducive to the onset of cardiac rhythm disorders [22]. In our cohort, overall mortality was 27.8%, rising to 35.6% in patients with a eGFR below 60 mL/min/1.73 m ^2^. Impaired kidney function doubled the risk of death, and survival was poorer in this group. These results are comparable to those reported in the literature [1,19,23,24]. Uribarri et al. reported a survival rate almost halved at 10 days (80.8% vs 41.4%) [1]. Trabulus et al. in their single-center study of 336 patients reported that eGFR < 60 mL/min/1.73 m^2^ was a predictor of mortality with a reduced survival rate (OR 2.61 CI: 1.121–4.167) [19]. Better still, Suk et al. in a retrospective study reported that the optimal eGFR cutoff value was 56 mL/min/1.73 m^2^ for predicting in-hospital mortality in pneumonia [25]. Mortality increased with the degree of kidney function impairment, with a greater risk of death for kidney function at admission ≤ 29 mL/min/1.73 m ^2^ [26,27]. In our study, an increased risk was progressively observed as admission eGFR values declined, reaching its highest level at less than 15 mL/min/1.73 m^2^ (Supplementary Table 1). This linear relationship between admission eGFR and mortality was not always reported. In a retrospective study and utilizing fractional polynomial modeling, Ayubi et al. reported that the risk of mortality in COVID-19 patients increases exponentially in eGFR < 45 mL/min/1.73 m ^2^, increases linearly in eGFR 45 to 60 mL/min/1.73 m ^2^ and after that tends to plateau [28].

Stratification of patients at risk of death remains a priority in order to reduce the burden associated with critical illness, especially as early determination of the severity of the disease improves its outcome [25]. This study has the advantage of adding to the markers of kidney damage (proteinuria and AKI) studied in the same environment [7,9], eGFR on admission as a factor associated with mortality. This marker offers a real advantage in our setting, where the diagnosis of AKI is often difficult due to the lack of repeated serum creatinine measurements. Zhang et al. using a cutoff of 82.2 mL/min/1.73 m ^2^, showed that even slight alterations in kidney function were associated with disease progression and therefore mortality [29].

The risk of mortality associated with kidney failure varies with several factors. Based on the existing study on the association between eGFR and outcome, Liu et al. reported that age modifies the association between eGFR and mortality [30]. In an observational study of 123 COVID-19 patients, Longhitano et al. reported that hypernatremia increased the risk of mortality associated with kidney failure [31]. The influence of hypoxia on mortality in patients with kidney failure has not been widely reported, and our results may suggest that these two markers of organ failure (respiratory and kidney) are separately predictive of mortality on the one hand, and can therefore be included in a risk stratification model. On the other hand, this association may be reminiscent of the pathogenic interaction between the lung and the kidney, known as kidney-lung organ cross-talk [6]. Although COVID-19 is primarily a respiratory disease, inflammatory mediators can create a viscous cycle between acute lung injury (ALI) and acute kidney injury, leading to the death of COVID-19 patients. Kulvichit et al. studied the association of different patterns of AKI and acute respiratory failure (ARF) with mortality. In a prospective multi­­center study, these authors reported higher in-hospital mortality in patients with dual organ failure (53% in “ARF first” and 54.4% in “AKI first” and concurrent AKI-ARF (69.9%) defined by development of AKI and ARF on the same day, within 24h) [5]. They highlighted the role of fluid overload as a major independent risk factor for the development of secondary organ failure in both AKI after ARF and ARF after AKI [5].

KD and hypoxia were separately predictive of mortality despite the availability of support therapy. Although the design of our study does not allow us to address this finding, we can speculate on some limitations in the management of severe or critically ill patients in our setting, where intermittent hemodialysis (not recommended for patients with multisystem failure) was the only hemodialysis modality, where certain monitoring parameters were not repeated, where the decision to use mechanical ventilation was made too late, with a lengthy process of switching from one respiratory support modality to another, and where bacterial superinfection was not negligible.

Patients with concomitant KD and hypoxia on admission were older, had an inflammatory profile and more comorbidities such as hypertension and heart failure, all of which may explain the risk of death. Despite these morbid associations, multivariate analysis showed that the association of KD and hypoxia was an independent predictor of death, demonstrating its large impact on the risk of death.

Our study has some limitations. First, the retrospective nature of the study means that several variables of interest are not available, making it impossible to better evaluate the model and examine the outcome of each model of simultaneous kidney and lung injury limited on the concurrent kidney-lung dysfunction. Second, the absence of baseline and follow-up eGFR values makes it impossible to distinguish between acute and chronic deterioration on the basis of eGFR at admission. Nevertheless, this study has the advantage of being the first in the DRC to report the association of kidney dysfunction on admission with mortality in patients hospitalized with COVID-19 and to highlight one of the kidney-lung cross-talk models in the context of SARS-CoV-2 infection.

## Conclusion

Impaired kidney function on admission and hypoxia are common in COVID-19 patients. This suggests a possible bidirectional relationship between lung and kidney impairment, which in turn are an independent predictor of mortality in COVID-19 patients. Mortality risk stratification of patients should integrate admission GFR estimation and peripheral oxygen saturation to define groups of patients for whom early and specific intervention should be considered.

## Supplementary Material

Supplemental Material

## Data Availability

The dataset supporting the conclusion of this article are available from the corresponding author on reasonable request.
