# Supplementary material for: The influence of hypoxia in predicting COVID-19 mortality in patients with kidney dysfunction on admission in 2 centers in sub-Saharan Africa
Source: Ren Fail. 2026 Aug 26;48(1):2719962. doi: 10.1080/0886022X.2026.2719962 (PMC13523106; doi:10.1080/0886022X.2026.2719962)
Supplement: Supplementary file 1 [file IRNF_A_2719962_SM7482.docx]

**Supplementary Table 1. Predictors of mortality in COVID-19 patient**

| Variables | Univariate analysis | | Multivariate analysis | | | | | | |
| --- | --- | --- | --- | --- | --- | --- | --- | --- | --- |
|  |  |  |  | Model 1 | | Model 2 | | Model 3 | |
|  | p | HR (CI 95%) |  | p | aHR (CI 95%) | p | aHR (CI 95%) | p | aHR (CI 95%) |
| Age≥65years |  |  |  |  |  |  |  |  |  |
| No |  | 1 |  |  | 1 |  | 1 |  | 1 |
| Yes | 0.009 | 1,.69(1.14-2.49) |  | 0.346 | 1.22(0.81-1.82) | 0.384 | 1.20(0.80-1.79) | 0.291 | 1.24(0.83-1.87) |
| Comorbidities |  |  |  |  |  |  |  |  |  |
| No |  | 1 |  |  | 1 |  |  |  |  |
| 1 | 0.108 | 1.51(0.91-2.49) |  | 0.289 | 1.31(0.79-2.17) |  | - |  | - |
| ≥2 | 0.001 | 2.27(1.40-3.69) |  | 0.065 | 1.61(0.97-2.65) |  | - |  | - |
| Hypertension |  |  |  |  |  |  |  |  |  |
| No |  |  |  |  |  |  | 1 |  | 1 |
| Yes | 0.017 | 1.63(1.09-2.44) |  |  | - | 0.630 | 1.11(0.73-1.69) | 0.36 | 1.22(0.80-1.86) |
| Obesity |  |  |  |  |  |  |  |  |  |
| No |  | 1 |  |  |  |  | 1 |  | 1 |
| Yes | 0.001 | 2.15(1.35-3.43) |  |  | - | **0.007** | 1.94(1.19-3.15) | **0.013** | 1.86(1.14-3.02) |
| Hypoxia + eGFR |  |  |  |  |  |  |  |  |  |
| ≥60 + no hypoxia |  | 1 |  |  |  |  |  |  | 1 |
| ≥60 + hypoxia | 0.683 | 1.26(0.42-3.76) |  |  | - |  | - | 0.554 | 1.39(0.46-4.19) |
| 59.9-45 + no hypoxia | **0.041** | 2.14(1.07-4.27) |  |  | - |  | - | 0.096 | 2.24(0.87-5.80) |
| 59.9-45 + hypoxia | **0.032** | 2.66(1.04-6.80) |  |  | - |  | - | **0.036** | 2.11(1.05-4.25) |
| 44.9-30 + no hypoxia | **0.005** | 3.57(1.47-8.69) |  |  | - |  | - | **0.011** | 3.20(1.31-7.86) |
| 44.9-30 + hypoxia | **0.005** | 3.89(1.52-9.95) |  |  | - |  | - | **0.003** | 3.44(1.53-7.75) |
| 29.9-15 + no hypoxia | **<0.001** | 4.19(1.90-9.25) |  |  | - |  | - | **0.006** | 3.82(1.46-10.01) |
| 29.9-15 + hypoxia | **<0.001** | 5.45(2.78-9.79) |  |  | - |  | - | **<0.001** | 4.13(2.76-7.98) |
| <15 + no hypoxia | **<0.001** | 5.79(3.07-9.94) |  |  | - |  | - | **<0.001** | 5.28(2.92-8.38) |
| <15 + hypoxia | **<0.001** | 6.73(3.15-9.98) |  |  | - |  | - | **<0.001** | 6.02(3.21-8.97) |
| eGFR (ml/min/1.73m²) |  |  |  |  |  |  |  |  |  |

Bold values are the statistically significant p defining the variables associated to mortality. eGFR estimated glomerular filtration rate
